# The effect of femtosecond laser fluence and pitches between V-shaped microgrooves on the dynamics of capillary flow

**DOI:** 10.1016/j.rinp.2020.103606

**Published:** 2020-12

**Authors:** Fei Xie, Jianjun Yang, Chi-Vinh Ngo

**Affiliations:** a The Photonics Laboratory, Changchun Institute of Optics, Fine Mechanics and Physics, Chinese Academy of Sciences, Changchun 130033, China; b University of Chinese Academy of Sciences, Beijing 100039, China

**Keywords:** Superwicking aluminum surface, V-shaped microgroove, Femtosecond laser ablation, Isotropic-anisotropic flow transition, Heat dissipation

## Abstract

Open microgroove is one kind of capillary-driven superwicking surface structure. In this study, arrays of parallel V-shaped microgrooves were fabricated on an aluminum surface by using a femtosecond laser to obtain a superwicking surface which can quickly transport the water uphill against gravity. The relationships between the flowing time and flowing distance were investigated and compared with theoretical results. We demonstrated both laser fluence and scanning step size can affect the superwicking performance. The aluminum surfaces fabricated at a laser fluence of 18.49 J/cm^2^ and 52.67 J/cm^2^ showed the best superwicking performances with the average water flow velocities approximately 16.2 mm/s and 16.4 mm/s, respectively, in the distance of 30 mm. On the other hand, the superwicking surfaces show an anisotropic flow characteristic due to the parallel microgrooves structure. However, when the scanning step size drops to 25 µm, the surface will form irregular rough structures that result in the isotropic flow characteristics. Moreover, by using a thermal camera, we found that after a 10 µL water droplet was dropped into the heated surface, the superwicking surface temperature quickly dropped from 92.4 °C to 82.5 °C which indicated that laser processing of the superwicking surface has potential application in heat dissipation.

## Highlights

• Excellent superwicking aluminum surface is successfully fabricated.
• Effects of laser fluence and step size on superwicking property are investigated.
• Micro/nanostructures can enhance superwicking performance.
• Irregular V-shaped microgrooves degrade superwicking performance.
• Potential application in heat dissipation is proposed.

## Introduction

Superwicking surfaces have been widely used in the researches of thermal management [1], [2], [3], water collection [4], [5], [6], microfluidic [7], [8] and biomedical devices [9], [10], [11], due to their unique characteristic of automatically transferring liquids. In nature, many plants and animals have existed this property which can assist them in obtaining water. For example, the surface of a Sarracenia trichome can harvest and transport water extremely fast through its specific hierarchical microchannel structures [5]. Spider silk also can capture and transport water by a unique fibres structure of the periodic spindle-knots and joints [6]. In general, performance of superwicking surfaces depends on wettability of a solid, which is mainly affected by surface energy, liquid viscosity and the morphology of the solid surface [12], [13]. Based on these factors, various techniques have been developed to intrinsically change the wettability of solid surfaces, such as plasma treatment [14], [15], chemical etching [16], [17], [18], mechanical treatment [2], [19], [20] and laser ablation [21], [22], [23], [24], [25], [26]. Among these methods, laser ablation technology has more advantages such as simple operation and no need for a complex processing environment. Laser ablation can process nearly all types of materials and control the surface wettability by changing laser processing parameters flexibly [12]. Vorobyev et al. used high-intensity femtosecond laser pulses to create an array of parallel microgrooves covered with nanostructures on metal [21], glass [22] and silicon[23] for producing excellent superwicking surfaces. Kai Yin et al. processed a line-patterned superwetting surface on sapphire by using a femtosecond laser, and they found that the performance of superwicking property is enhanced with the increase of the width and depth of the microgrooves structures [25]. HuaZhong Zhu et al. used a sub-nanosecond laser to modify a nickel surface, which significantly enhances the wicking property and reduces the contact angles from ~96° to ~3.1° [26].

At present, there are many studies on using a femtosecond laser to fabricate the superwicking surface [21], [22], [23], [24], [25], [27], but few on the influence of laser processed surface structures on dynamics of superwicking action [28], [29], [30]. Besides, the excellent dynamics performance of sucking the liquid uphill against gravity on a superwicking surface is very important to meet more application requirements. In this study, we fabricated an array of parallel microgrooves on aluminum surfaces by using a femtosecond laser. The fabricated surfaces showed a powerful capillary action. We compared the experimental results with a theoretical model which was proposed by Rye et al. [31], and the effects of laser fluence and microgrooves step sizes on the performance of the superwicking surfaces were investigated. Additionally, we explored a potential application of the laser processed superwicking surfaces in heat dissipation.

## Materials and methods

### Materials

Aluminum samples with a purity of 99.999% and with a thickness of 2 mm were cleaned by an ultrasonic cleaner with ethanol before laser treatment to remove any contaminants.

### Laser fabrication

**Fig. 1:**
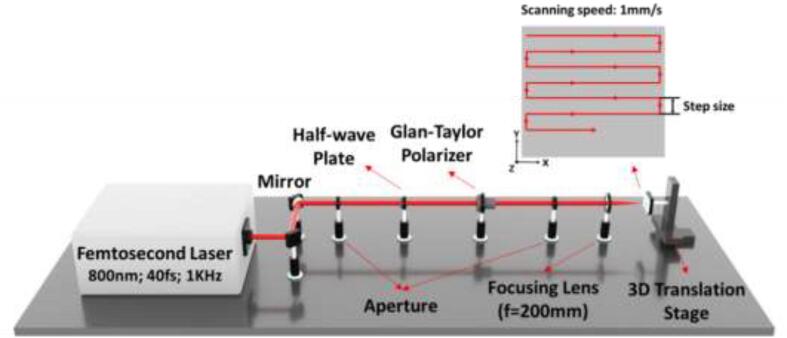
Femtosecond laser experimental setup for fabricating superwicking surfaces.

The as-prepared samples were fabricated by an amplified Ti:sapphire laser system which generates 40 fs pulses with a wavelength of 800 nm and repetition of 1 kHz. And the beam quality factor ($M^{2}$) of the laser is 1.3. A schematic diagram of the laser processing system is shown in Fig. 1. In this setup, a half-wave plate and a Glan-Taylor polarizer were used to control the laser fluence. A focusing lens (Focal length f = 200 mm) was used to focus the laser beam on the sample surface. The laser beam then was scanned on the sample surface by controlling a three-dimensional translation stage (LTA-HS, Newport), where the samples were fixed. By scanning on the sample surface line by line with the laser beam, arrays of parallel microgrooves with different step sizes were generated. The micro-grooves were processed under different fluence with a scanning speed of 1 mm/s. Table 1 shows the fabrication parameters of laser surface ablation. The arrays of micro-grooves were fabricated in an atmospheric environment. Additionally, the size of the fabrication area with different processing parameters was 2 mm × 33 mm. After laser fabrication, the samples were cleaned by an ultrasonic cleaner with deionized water to remove deposited particles during the processing and undesired contaminations.

**Table 1:** Fabrication parameters of laser surface ablation.

Laser fluence (J/cm^2^)	Step size (µm)
11.63	75
18.49	75
27.71	75
35.55	75
44.05	75
52.67	25, 75, 125
61.56	75
68.42	75

### Characterization

**Fig. 2:**
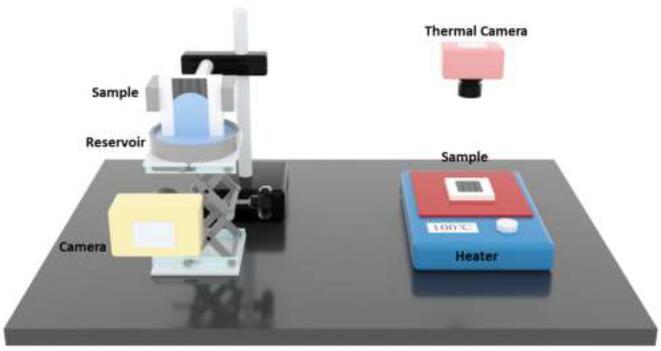
Schematic diagram of the setup for recording the water spreading process.

A scanning electron microscopy (SEM) and a three-dimensional (3D) laser confocal microscopy were used to analyze the surface morphology. A MultiFileAnalyzer software was used to obtain an average profile of the microgrooves and to measure the geometric parameters. The wetting property of the laser treatment samples was evaluated by measuring the contact angles of 1.5 µL water droplets with a contact angle measurement instrument. The samples were placed vertically by a holder, and a camera was used to record the liquid spreading process as shown in Fig. 2. To explore the potential application of the superwicking surface in heat dissipation, a heater was used to heat the laser processed sample and a thermal camera was used to measure the surface temperature.

## Results and discussions

### Surface morphology

**Fig. 3:**
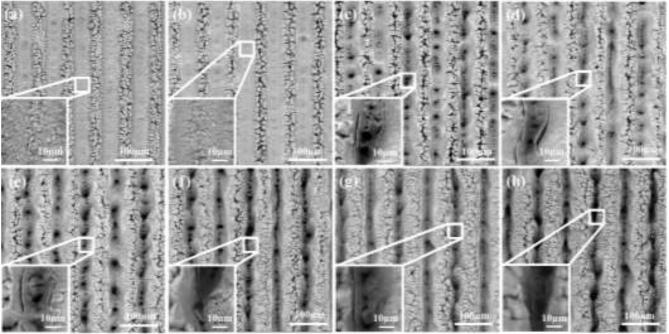
SEM images of the laser-treated aluminum surfaces with different laser fluence: (a) 11.63 J/cm^2^, (b) 18.49 J/cm^2^, (c) 27.71 J/cm^2^, (d) 35.55 J/cm^2^, (e) 44.05 J/cm^2^, (f) 52.67 J/cm^2^, (g) 61.56 J/cm^2^, (h) 68.42 J/cm^2^.

SEM images of the aluminum surfaces illustrated an appearance of multiple parallel microgrooves under different laser fluence with the same step size of 75 µm, as shown in Fig. 3. The surface-treated at 11.63 J/cm^2^ and 18.49 J/cm^2^, as shown in Fig. 3(a) and (b), appeared the regular V-shaped microgrooves which are extensively covered with irregular nanocavity and nanoprotrusion structures. As the fluence of laser pulses increased, the depth of microgrooves became greater and additional micro-cavities and micro-cracks were generated in the valleys and ridges of the microgrooves. On the one hand, due to the high laser fluence, more materials were removed and more micro/nanoparticles were deposited along the microgrooves. Moreover, the high pulse overlap rate leads to the heat accumulation effect [32], [33] which causes the material to melt and makes the shape of the microgrooves changed from regular V-shaped into irregular V-shaped. When the laser fluence is in the range of 27.71 J/cm^2^ to 44.05 J/cm^2^, as shown in Fig. 3(c)-(e), the valley surfaces are full of isolated microbumps and micropores because the micro/nanoparticles deposited on the surface affect the removal of materials and heat accumulation effect lead to inhomogeneous structures. These isolated microbumps and micropores were connected by smaller micropores at the laser fluence of 52.67 J/cm^2^, which led to the deeper microgrooves. Although the microgrooves became extremely deep at the laser fluence of 68.42 J/cm^2^, the shape of the microgrooves became more irregular due to more deposited micro/nanoparticles and stronger heat accumulation effect that comes from the high laser fluence and high pulse overlap rate.

**Fig. 4:**
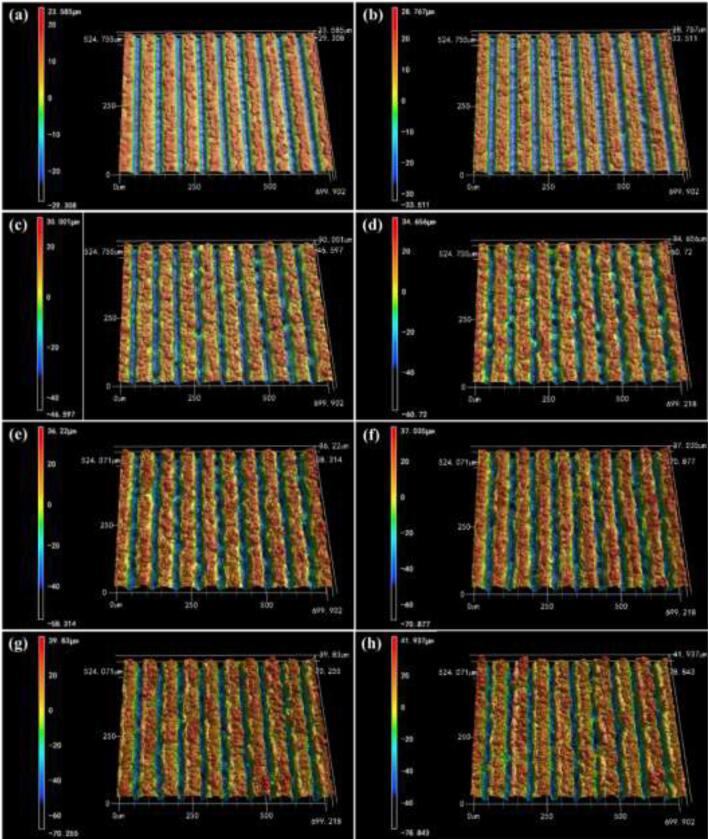
3D images of the laser treated aluminum surfaces with different laser fluence: (a) 11.63 J/cm^2^, (b) 18.49 J/cm^2^, (c) 27.71 J/cm^2^, (d) 35.55 J/cm^2^, (e) 44.05 J/cm^2^, (f) 52.67 J/cm^2^, (g) 61.56 J/cm^2^, (h) 68.42 J/cm^2^.

3D images and the geometric parameters such as the depth and angle of the microgrooves were analyzed with a three-dimensional (3D) laser confocal microscopy, as showed in Fig. 4. The average value of microgrooves geometric parameters were used to characterize the physical dimension of V-shaped micro-structures under different processing parameters. Table 2 shows the measurement results and a schematic diagram of the V-shaped microgrooves.

**Table 2:** The average angle and depth of V-shaped microgrooves under different laser fluence.

Schematic diagram of V-shaped structure	Laser fluence (J/cm^2^)	Angle β (°)	Depth d (µm)
	11.63	66.55	35.45
	18.49	58.16	39.64
	27.71	55.52	43.10
	35.55	53.38	46.13
	44.05	48.04	53.20
	52.67	40.21	62.50
	61.56	35.89	63.17
	68.42	33.92	61.68

### Water contact angle measurement

**Fig. 5:**
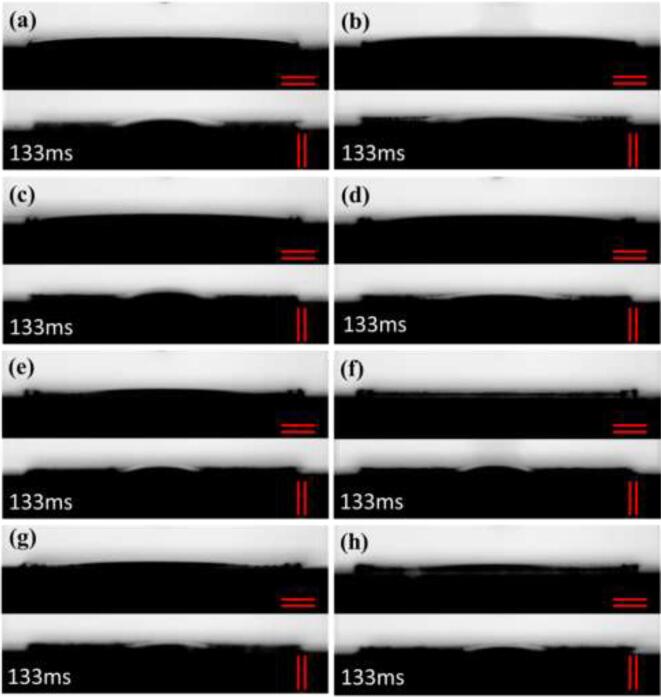
The results of 1.5 µL water droplets spread in vertical (||) and parallel (=) microgrooves directions on the surface treated under different laser fluence: (a) 11.63 J/cm^2^, (b) 18.49 J/cm^2^, (c) 27.71 J/cm^2^, (d) 35.55 J/cm^2^, (e) 44.05 J/cm^2^, (f) 52.67 J/cm^2^, (g) 61.56 J/cm^2^, (h) 68.42 J/cm^2^. (The red lines represent the microgrooves direction.) (For interpretation of the references to colour in this figure legend, the reader is referred to the web version of this article.)

When the 1.5 µL water droplet was dropped into the surface, the water droplet quickly spread on all of the sample's surface with a contact angle close to 0°. This means that the surface changed from hydrophilic to superhydrophilic properties. To compare the flowing state of water droplets along with the vertical and parallel microgroove directions, the moment when a water droplet touches the surface is defined as 0 ms. Fig. 5 shows the flowing states of the water droplet flowed along vertical and parallel the fabrication direction of microgrooves at 133 ms. The samples under all processing parameters show that the velocity of the liquid flowing along the microgroove is much higher than that of droplet spread in the direction vertical to the microgroove. It indicates that water droplets flow preferentially along the microgroove direction and the laser-treated surfaces show an anisotropic flow characteristic. The parallel microgrooves structure leads to this anisotropic flow characteristic due to the stronger capillary force along the microgroove direction.

### Wicking performance

Capillary force plays an important role in driving the water spread along the microgrooves on wicking surfaces. The viscous friction force coming from the liquid is the main flowing resistance. Following the model of liquid spread in V-shaped microgrooves proposed by Rye et al.[31], the capillary force ($F_{\mathrm{cap}}$) and the viscous friction force ($F_{\mathrm{vis}}$) can be expressed as:

(1) $$F_{\mathrm{cap}}=\frac{2d\gamma }{\sin\left(\alpha \right)}\left[\cos\left(\theta \right)-\frac{\left(\alpha -\theta \right)}{\sin\left(\alpha -\theta \right)}\cos\left(\alpha \right)\right]$$

(2) $$\alpha =90{}^{\circ}-\frac{\beta }{2}$$

(3) $$F_{\mathrm{vis}}=8\pi \mu h\frac{\mathrm{dh}}{\mathrm{dt}}$$

where d and β are the depth and the angle of the V-shaped microgrooves, respectively. And the θ is the static advancing contact angle (CA) of the wicking surface. γ and μrepresent the surface tension ($72.28\times 10^{-3}N/m$) and viscosity ($0.9358\times 10^{-3}Pa\cdot S$) of water. h and t represent the flow liquid front position and spread the time of liquid in the microgrooves.

In this model, they use a simple force balance between the net Newtonian force, the surface capillary driving force (F_cap_) and the viscous friction retarding force (F_vis_) to obtain the general relation:

(4) $$m\frac{d^{2}h}{dt^{2}}=F_{\mathrm{cap}}-F_{\mathrm{vis}}$$

integrating the Eq. (4) can further obtain the kinetic expression:

(5) $$h^{2}=\frac{1}{2\pi \sin\left(\alpha \right)}\left[\cos\left(\theta \right)-\frac{\left(\alpha -\theta \right)}{\sin\left(\alpha -\theta \right)}\cos\left(\alpha \right)\right]\frac{\gamma d}{\mu }t$$

**Fig. 6:**
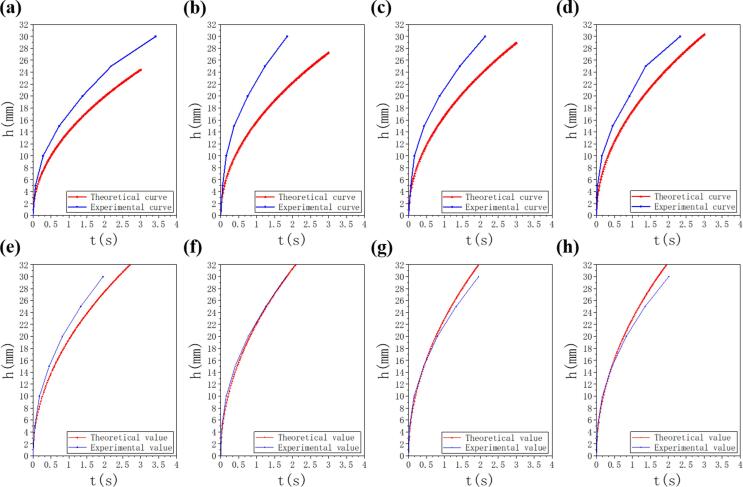
Comparison of experimental (blue) and theoretical (red) curves of the relationships between the flowing time and flowing distance of different samples treated with laser fluence: (a) 11.63 J/cm^2^, (b) 18.49 J/cm^2^, (c) 27.71 J/cm^2^, (d) 35.55 J/cm^2^, (e) 44.05 J/cm^2^, (f) 52.67 J/cm^2^, (g) 61.56 J/cm^2^, (h) 68.42 J/cm^2^. (For interpretation of the references to colour in this figure legend, the reader is referred to the web version of this article.)

Base on this model, using the Eq. (5) and the physical dimension of the V-shaped microgrooves in Table 2 to obtain the theoretical relationships between the flowing time and flowing distance on the surfaces with different laser fluence which are compared with experimental curves showed in Fig. 6.

As seen in Fig. 6, when the laser fluence is lower than 44.05 J/cm^2^, the experimental curves are higher than the theoretical curves because the nanostructures produced by laser processing enhance the capillary. With the laser fluence increasing greater than 18.49 J/cm^2^, the experimental curves are gradually close to or even lower than the theoretical curves because the more deposited micro/nanoparticles and heat accumulation effect make the shape of the microgrooves change from regular to irregular, which may increase friction force or weaken capillary force. In the case of laser fluence between 11.63 J/cm^2^ and 18.49 J/cm^2^, the treated surfaces form regular V-shaped microgrooves covered with nanocavities and nanoprotrusions due to the nanoparticles deposited during laser processing. These nanostructures make the surface rougher and increase the ratio of actual surface area to the geometric area which can enhance superwicking performance by increasing the driving capillary pressure. Microgrooves on aluminum surface fabricated at 18.49 J/cm^2^ have greater depths (d) and lower angles (β) than those of microgrooves on aluminum surface fabricated at 11.63 J/cm^2^, so this surface has more nanostructures and a strong capillary force, resulting in higher superwicking performance. However, when the laser fluence rises to 27.71 J/cm^2^, the valley surfaces are full of isolated microbumps and micropores. The water is easily absorbed into and difficultly flow out of the structures which obstructs the water flow. Additionally, deposited micro/nanoparticles and heat accumulation effect coming from the high pulse overlap rate will make V-shaped microgrooves irregular. The irregular microgrooves structure results in irregular cross-sectional area changes, which further affects the driving capillary pressure, and the theoretical model is not applicable properly. These two factors gradually counteract the enhancement of nanostructure on the superwicking property with the increase of laser fluence, which makes the experimental curve close to the theoretical curve and even lower than it.

**Fig. 7:**
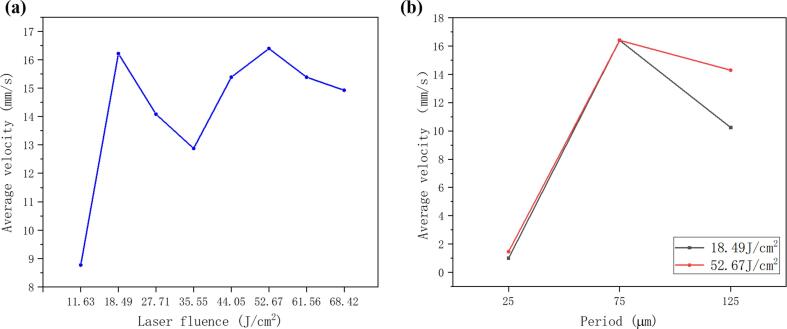
The average water flowing velocity of the first 30 mm of the superwicking surfaces treated with (a) different laser fluence and (b) different step sizes.

The average water flowing velocity of the first 30 mm of the surface treated with different laser fluence were compared in Fig. 7(a). From the results, the samples fabricated at 18.49 J/cm^2^ and 52.67 J/cm^2^ show the best superwicking performances with the average water flow velocities roughly 16.2 mm/s and 16.4 mm/s, respectively, in the distance of 30 mm. For the sample fabricated at 18.49 J/cm^2^, its regular V-shaped microgrooves have smaller friction resistance and the nanostructures covered on the surface of microgrooves provide strong capillary force. Therefore, the sample fabricated at 18.49 J/cm^2^ can quickly transport liquid uphill against gravity. For the sample fabricated at 52.67 J/cm^2^, it shows similar superwicking performance to that of the sample fabricated at 18.49 J/cm^2^ due to its deeper microgrooves and smaller angle microgroove structures, although the irregular V-shaped microgrooves including micro-cavities and micro-cracks generated in the valleys and ridges of the microgrooves have high friction resistance which counteracts the enhancement of nanostructure on the superwicking property. The cross-sectional area of the sample fabricated at 52.67 J/cm^2^ is larger than that of the sample fabricated at 18.49 J/cm^2^. Consequently, during the same time, the sample fabricated at 52.67 J/cm^2^ can transport more volume of water which is about 1.5 times the sample fabricated at 18.49 J/cm^2^.

**Fig. 8:**
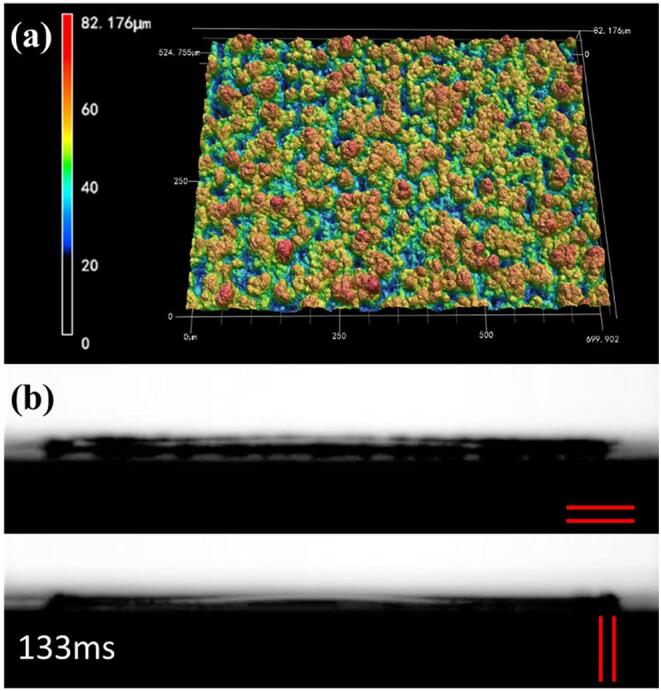
(a) The 3D image of the sample with a step size of 25 µm. The results of a 1.5 µL water droplet spread in vertical (||) and parallel (=) microgrooves directions on the surface.

The femtosecond laser pulses with powers of 52.67 J/cm^2^ were used to process arrays of parallel micro-grooves on the aluminum surface with different step sizes of 25 µm, 75 µm and 125 µm, respectively. The average velocities of water flowing on the surfaces prepared by different processing parameters were compared in Fig. 7(b). The average velocity of the samples with a step size of 25 µm was far less than that of the sample with a step size of 75 µm and 125 µm due to the different structures. The 3D image of the sample with a step size of 25 µm showed in Fig. 8(a), the parallel microgrooves structure disappeared, and an irregular rough surface was formed due to the strong overlap between two adjacent microgrooves. Although these irregular rough structures still could suck the water uphill, they greatly reduced the capillary force in the laser scanning direction. As seen from Fig. 8(b), the water droplet showed similar flow behavior along the scanning direction and vertical scanning direction which indicates that the water droplet rapidly spread around isotropically on the irregular rough surface.

### Thermal performance

**Fig. 9:**
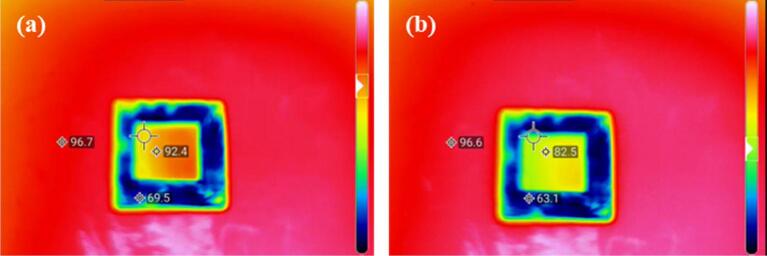
The IR image of the superwicking surface. (a) Keep heating until the temperature is stable. (b) After a 10 µL water droplet was dropped into the superwicking surface.

Fig. 9 shows the temperature of the heater surface (96.7 °C), the raw area of the sample (69.5 °C) and the laser processing area (92.4 °C) after heating. This result shows that the laser processed surface has excellent heat dissipation performance. When a 10 µL water droplet was dropped into the laser processed area, the water droplet quickly spread and form a thin film of water on the surface due to its superwicking property which can enhance water evaporation and further lead to the surface temperature of the laser processed area is reduced by about 10 °C. Consequently, the superwicking surface processed by femtosecond laser could be applied in heat dissipation.

## Conclusion

In this work, an excellent superwicking surface was processed on aluminum by using a femtosecond laser. The dynamic performance of transporting the water uphill against gravity on the superwicking surfaces was investigated varying the different processing parameters. The results show that both laser fluence and scanning step size will affect superwicking performance. The nanostructures covered on the microgrooves can effectively enhance superwicking performance. As the laser fluence increases, the shape of the microgrooves became more irregular due to more deposited micro/nanoparticles and the heat accumulation effect that comes from the high laser fluence and pulse overlap rate. The aluminum surfaces fabricated at 18.49 J/cm^2^ and 52.67 J/cm^2^ show the best superwicking performances with the average water flow velocities approximately 16.2 mm/s and 16.4 mm/s, respectively, in the distance of 30 mm. During the same time, the surface fabricated at 52.67 J/cm^2^ can transport more volume of water (approximately 1.5 times) than that of the surface fabricated at 18.49 J/cm^2^. Due to its larger cross-sectional area. The parallel microgrooves structure leads to an anisotropic flow characteristic, however, when the scanning step size drops to 25 µm the surface will form an irregular rough structure which results in the isotropic flow characteristics. Therefore, the key to obtaining a high-performance superwicking surface is to process a deeper and regular microgroove with a smaller angle of the structures covered with nanostructures. By using a thermal camera, we found that when a 10 µL water droplet was dropped into the heated surface, the water droplet quickly spread and form a thin film of water on the superwicking surface, which can enhance water evaporation and further lead to a decrease of surface temperature. Besides, due to the ability to faster transport liquid uphill against gravity, the superwicking surfaces can be widely used in the evaporative cooling system, water collection, liquid directional transport and other potential applications.

## CRediT authorship contribution statement

**Fei Xie:** Investigation, Writing - original draft. **Jianjun Yang:** Writing - review & editing. **Chi-Vinh Ngo:** Conceptualization, Writing - review & editing.

## Declaration of Competing Interest

The authors declare that they have no known competing financial interests or personal relationships that could have appeared to influence the work reported in this paper.
